# LINE1 family member is negative regulator of HLA-G expression

**DOI:** 10.1093/nar/gks874

**Published:** 2012-09-21

**Authors:** Masashi Ikeno, Nobutaka Suzuki, Megumi Kamiya, Yuji Takahashi, Jun Kudoh, Tsuneko Okazaki

**Affiliations:** ^1^School of Medicine, Keio University, Shinanomachi, Shinjuku-ku, Tokyo 160-8582, ^2^Chromo Research Inc., 1212 Shihongi, Midori-ku, Nagoya, Aichi 458-0039 and ^3^Institute for Comprehensive Medical Science, Fujita Health University, Toyoake, Aichi 470-1192, Japan

## Abstract

Class Ia molecules of human leucocyte antigen (HLA-A, -B and -C) are widely expressed and play a central role in the immune system by presenting peptides derived from the lumen of the endoplasmic reticulum. In contrast, class Ib molecules such as HLA-G serve novel functions. The distribution of HLA-G is mostly limited to foetal trophoblastic tissues and some tumour tissues. The mechanism required for the tissue-specific regulation of the HLA-G gene has not been well understood. Here, we investigated the genomic regulation of HLA-G by manipulating one copy of a genomic DNA fragment on a human artificial chromosome. We identified a potential negative regulator of gene expression in a sequence upstream of HLA-G that overlapped with the long interspersed element (LINE1); silencing of HLA-G involved a DNA secondary structure generated in LINE1. The presence of a LINE1 gene silencer may explain the limited expression of HLA-G compared with other class I genes.

## INTRODUCTION

Class Ia molecules of human leucocyte antigen (HLA-A, -B and -C) and class Ib molecules (HLA-E, -F and -G) are members of human major histocompatibility complex (MHC), a cell surface molecule encoded by gene family. Class Ia molecules are widely expressed in tissues and play a central role in the immune system by presenting peptides derived from the lumen of the endoplasmic reticulum (1). In contrast, the class Ib molecule HLA-G contributes to maternal tolerance of the allogeneic foetus and also to novel functions (2,3). Therefore, the distribution of HLA-G is mostly limited to foetal trophoblastic tissues and numerous tumour tissues (4,5).

HLA family genes were derived from gene duplication, accompanied by the insertion of retrotransposons (6,7). Although the sequences upstream (5′) and downstream (3′) of the coding region are analogous among HLA genes, they are often interrupted by highly abundant retrotransposons, including long interspersed elements (LINE1 or L1) (8,9). Previously, the HLA-G promoter was reported to be located in a region upstream of HLA-G that was analogous to the upstream regions of class Ia genes, with some polymorphisms (10,11). However, tissue-specific regulation of the HLA-G gene is not well understood.

Transgene expression in stable cell lines is often repressed by unexpected epigenetic silencing around the integrated position, complicating the search for regulatory elements. The human artificial chromosome (HAC), which replicates and segregates once per cell division cycle to be stably maintained in cells, is an alternative platform of gene expression (12). We previously developed a HAC vector system in which one copy of the DNA fragment of interest can be manipulated by Cre/lox insertion in any cell line of interest (13). Compared with viral or integrating vectors, this HAC vector is advantageous for evaluating gene expression without unexpected gene silencing.

Here, we used a HAC vector to investigate regulation of the HLA-G gene. With this approach, we identified a negative regulator of gene expression in a sequence upstream of HLA-G that overlapped with a LINE1 sequence.

## MATERIALS AND METHODS

### Cell culture

HT1080 cells and mouse embryonic fibroblast (MEF) cells were cultured in Dulbecco’s modified Eagle’s medium (Sigma) supplemented with 10% foetal bovine serum at 37°C and 5% CO_2_. JEG3 cells were cultured in Eagle’s minimal essential medium (Sigma) supplemented with 10% foetal bovine serum at 37°C and 5% CO_2_. Cell lines that harboured a HAC vector were selected with G418 (Sigma) at 400 µg/ml.

### HLA-G gene constructs

HLA-G cDNA was generated by reverse transcriptase–polymerase chain reaction (RT–PCR) and fused to the cytomegalovirus (CMV) promoter derived from pEGFP-C1 (Clontech) (CMV-HLA-G). A fragment of DNA carrying CMV-HLA-G or the 5.9-kb HLA-G genomic fragment (14) (HLA-G4) was cloned into the HindIII site of pLox66-puro vector (13) (pLox/CMV-HLA-G and pLox/HLA-G4, respectively). To extend the 5′ region of HLA-G4, we amplified a 5.8-kb sequence of the HT1080 genomic DNA with primers 5′-GCAATTGTGACAGAGAGGACCACGAGGCCATG-3′ and 5′-CTGCAAAGAACACCCAGCGAGGCTC-3′. The resulting DNA product was digested with MfeI or PmeI and ligated into the EcoRI (compatible with the MfeI site) and PmeI sites in the pLox/HLA-G4 construct, which contained 11.4 kb of the HLA-G genomic sequence. We deleted the 5′ region of HLA-G1 by digestion with I-CeuI (vector) and NheI or SnaBI (HLA-G), followed by self-ligation to produce pLox/HLA-G2 (10.1-kb HLA-G) or pLox/HLA-G3 (8.7-kb HLA-G), respectively.

### ****EGFP reporter constructs

The HLA-G minimal promoter (1.2 kb) and the human β-actin promoter (1.1 kb) were generated from HT1080 genomic DNA by PCR with primers HLA1 (5′-GGAGGTGAGGAAAAGGAGCAGAGG-3′) and HLA2 (5′-GACTCATTCTCCCCAGACGCCAAGG-3′) and hActF2 (5′-ATAGAATTCGCACATGGAGTCTTGGTCCCCAGAG-3′) and hActR1 (5′-ATAAAGCTTCGGACGCGGTCTCGGCGGTGGTGGC-3′), respectively. The PCR products were then cloned into the pDrive vector (QIAGEN). The EF1α promoter was derived from the pTracer vector (Invitrogen). These promoters replaced the CMV promoter at the AseI (blunting) and NheI sites of EGFP-C1 to produce the HLA-EGFP, Act-EGFP and EF1-EGFP constructs.

The 5.9-kb HLA-G fragment was digested with NheI and SnaBI to produce sequence gL1. The other LINE fragments were generated by PCR of HT1080 or TIG1 genomic DNA. For gL2, the PCR primers were gL1-1 (5′-TCCTATAGCCAGAAGAATCCTAAGC-3′) and gL1-4 (5′-AGAGAGCTGTACTAAAGGACTTACG-3′). The gL3 sequence was generated by PCR with primers gL1-1 and gL1-2 (5′-AAGCTCTTTTGTTTAATTAGATCC-3′). For gL4, the PCR primers were gL1-3 (5′-GTAGAGTGAAAGAAACTATTATCAG-3′) and gL1-4. The L1A sequence resulted from PCR with primers L1-1 (5′-ACTAAAATCAGAGCAGAACTGAAGG-3′) and L1-2 (5′-GTTCCATATGAACTTTAAAGTAGTTTTTTCC-3′). Sequence L1B was generated by PCR with primers L1-3 (5′-GAACCAAAAAAGAGCCCGCATTGCC-3′) and L1-4 (5′-GTTACATATGTATACATGTGCCATGCTGG-3′).

Each LINE fragment was inserted into the AseI site at the 5′-end of the CMV-EGFP sequence in pEGFP-C1. These LINE-CMV-EGFP cassettes were digested with AseI and MluI, then blunt-end ligated into the EcoRI site of the pLox66-puro vector. The gL1 fragment was inserted into a SnaBI site at the 5′-end of HLA-EGFP, and gL1-HLA-EGFP cassettes were also cloned into the EcoRI site of the pLox66-puro vector.

### gL deletion constructs of HLA-G

The gL element in the pLox/HLA-G1 construct was deleted by digestion with HpaI and SnaBI, and self-ligated to produce gL deletion constructs of HLA-G (ΔgL). The aL element was generated by PCR of HT1080 genomic DNA with primers aL-a (5′-ATCAGATTAGGCAATTTTTTTCTGCACAGC-3′) and aL-b (5′-ATTATTTTTCATTAACTCTTTAAGCTTGTC-3′). The gL element was generated by PCR of HT1080 genomic DNA with primers gL-a (5′-AACACCTAAGGCTCTGTGATGTCTC-3′) and gL-b (5′-GAGAGGGAATTAAGCTGCACCTCTG-3′). Constructs were generated that contained ΔgL plus the aL element (the SfcI–HindIII fragment) in the forward direction (ΔgL + aL), the gL element (the AseI–SnaBI fragment) in the reverse direction [ΔgL + gL(R)] or the gL element at a distal position [ΔgL + gL(dis)].

### DNA transfection

For insertion of the constructs into the HAC vector, 1 µg of the pLox66-puro vector containing the HLA-G gene or the EGFP expression cassette was co-transfected with 0.5 µg of CAGGS-Cre into HT1080, MEF or JEG3 cells (5 × 10^5^) with lipofectamine2000 (Invitrogen) or FuGENE HD (Roche) according to the manufacturer’s instructions. Cell lines were selected with puromycin (Sigma) at 0.25 µg/ml (HT1080), at 2 µg/ml (MEF) or at 0.5 µg/ml (JEG3).

### RT–PCR

Total RNA was isolated with the SV Total RNA Isolation System (Promega). cDNA was synthesized using the Transcriptor First Strand cDNA Synthesis Kit (Roche Applied Science); 25 ng aliquots were used for PCR. The EGFP gene was amplified with PCR primers EGFP1 (5′-CGACGTAAACGGCCACAAGTTCAG-3′) and EGFP2 (5′-CAGGACCATGTGATCGCGCTTCTC-3′). The PCR protocol was: 95°C for 4 min and 23 cycles of 95°C for 15 s, 68°C for 15 s, and 72°C for 30 s. For the HLA-G gene, the primers were HLA-G1 (5′-AGGCGGCCAATGTGGCTGAACAAA-3′) and HLA-G2 (5′-CAGGGTGGCCTCATAGTCAAAGACA-3′), and the PCR protocol was 95°C for 4 min and 23 cycles of 95°C for 15 s, 68°C for 15 s, and 72°C for 30 s.

### Western blot

Whole cell extracts were separated by 12.5% SDS–PAGE and transferred to polyvinylidene difluoride membranes (Millipore). Anti-HLA-G antibody (1:500 dilution; MBL) was used for immunodetection. Images were captured and detected with a LAS3000.

### Mung bean nuclease cleavage and PCR

Genomic DNA (200 ng) was cleaved with 5, 10 or 20 U of mung bean nuclease (MBN) (NEB) in a 20 µl reaction mixture at 30°C for 1 h. The LINE (gL2, L1A and L1B) in EGFP reporter cassettes, the LINE (gL and aL elements) in genome, HLA-A promoter, HLA-G promoter, β-actin promoter and EF1α promoter samples were amplified via 30 cycles of 94°C for 15 s, 60°C for 15 s, and 72°C for 60 s. CMV and EGFP sequences were amplified via 30 cycles of 94°C for 15 s, 65°C for 15 s and 72°C for 30 s. For gL2, the primers were gL2-1 (5′-TCCTATAGCCAGAAGAATCCTAAGC-3′) and CMV-Nr (5′-TATGGGCTATGAACTAATGACCCCG-3′). For L1A, the primers were LA1 (5′-AATCCAGGAGCTGGTTTTTTGAAAGG-3′) and CMV-Nr and for L1B, the primers were LB1 (5′-ACAAAGCTGGAGGCATCACACTACC-3′) and CMV-Nr. For gL element, the primers were gL1-a and gL1-b, and for aL element, the primers were aL1-a and aL1-b. For CMV, the primers were CMV1 (5′-TAATCAATTACGGGGTCATTAGTTC-3′) and CMV2 (5′-TGAACTTGTGGCCGTTTACGT-3′). Primers EGFP1 and EGFP2 were used for amplification of EGFP. The primers used for amplification of the HLA-A promoter were HLA-Ac (5′-TACATCAATCTACAGAGCCTAGGAG-3′) and HLA-Aa (5′-CCTCGGCGTCTGGGGAGAATCTGAG-3′), whereas primers HLA-Ga (5′-GACCGCGGCGACGCTGATTGGCTTCTC-3′) and HLA-Gc (5′-TATGTGGGTCTGCCTAGAAACTAATTG-3′) were used for the for HLA-G promoter. The actin promoter was amplified with primers hActF2 (5′-ATAGAATTCGCACATGGAGTCTTGGTCCCCAGAG-3′) and hActR1 (5′-ATAAAGCTTCGGACGCGGTCTCGGCGGTGGTGGC-3′). Primers EF1b (5′-CTGGGAAAGTGATGTCGTGTACTGG-3′) and EF1a (5′-TTTGAACCACTGTCTGAGGCTTGAG-3′) were used to amplify the EF1α promoter.

## RESULTS

### Analysis of HLA-G regulation using the HAC vector

**Figure 1.**
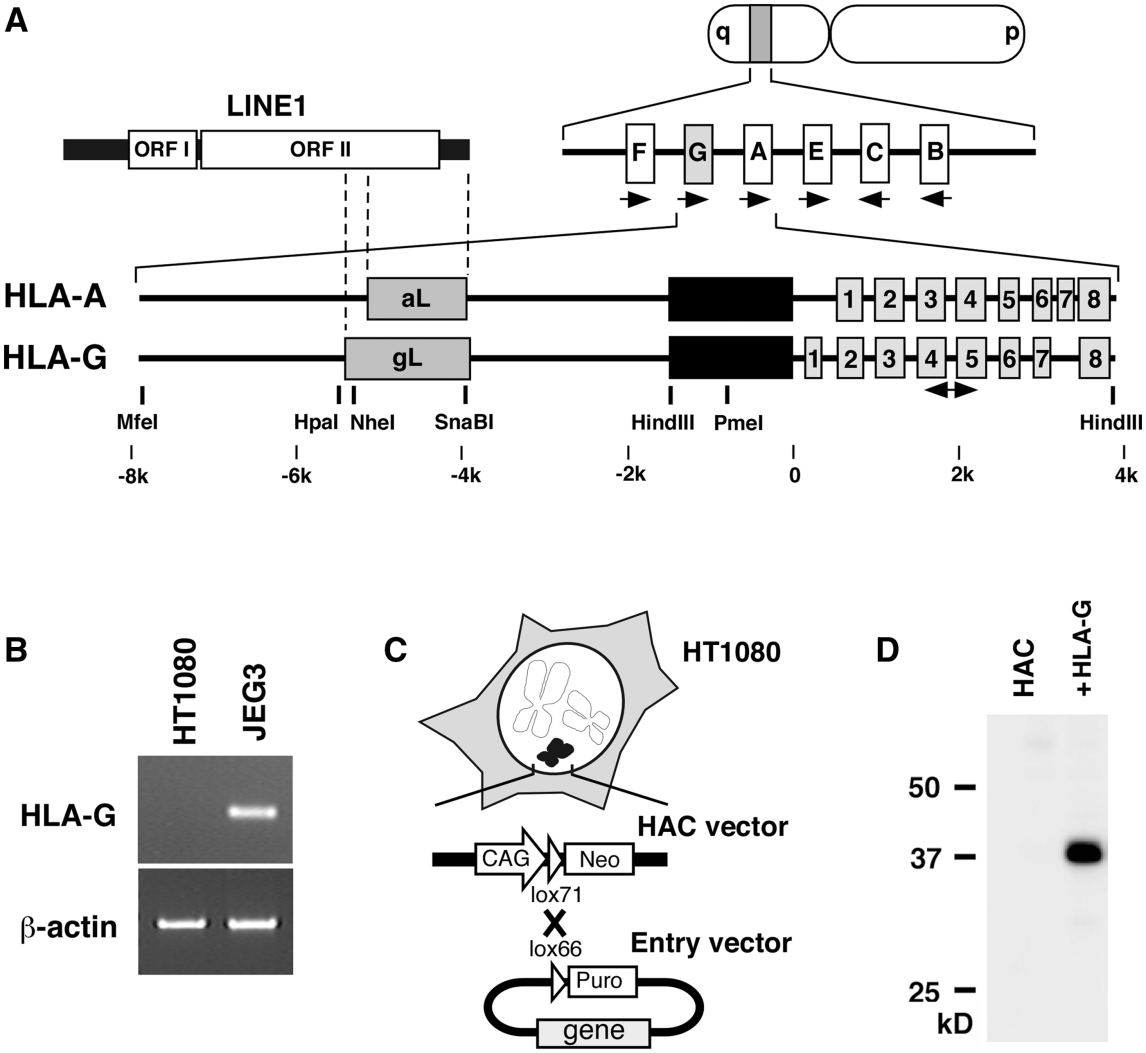
The HLA-G gene and the HAC vector. (**A**) Schematic representation of the HLA gene cluster. The HLA-A and HLA-G genes are composed of eight exons. Black boxes indicate the reported unique promoter regions; grey boxes indicate 3′ region of LINE1 family insertions. The two-way arrow indicates the position for the detection of HLA-G expression by RT–PCR. (**B**) Expression of HLA-G examined by RT–PCR. A 150-bp fragment of exons 4–5 was detected in RNA from JEG3 cells, but not in RNA from HT1080 cells. (**C**) Schematic representation of the HAC vector. The gene in the entry vector was inserted into a HAC vector in HT1080 cells by Cre/lox recombination. (**D**) The HLA-G product was detected by western blot with an anti-HLA-G antibody. Lane HAC is a sample from an HT1080 cell line harbouring the HAC vector, whereas the sample in lane +HLA-G came from a cell line in which construct CMV-cHLA-G was incorporated into the HAC vector. The 40-kD HLA-G protein was detected in the cell line expressing the HLA-G transgene.

To examine the tissue-specific expression of HLA-G, transcripts from cell lines were measured by RT–PCR with HLA-G-specific primers (Figure 1A). HLA-G was repressed in HT1080 cells derived from a fibrosarcoma, but was expressed in JEG3 cells from a placental source (Figure 1B). To investigate regulation of the HLA-G gene, we manipulated one copy of various genomic fragments using the HAC vector in HT1080 cell lines (13) (Figure 1C). The product of the HLA-G transgene was detected in HT1080 cells via western blotting with anti-HLA-G antibodies after introduction of a CMV-driven HLA-G cDNA using the HAC vector (Figure 1D).

**Figure 2.**
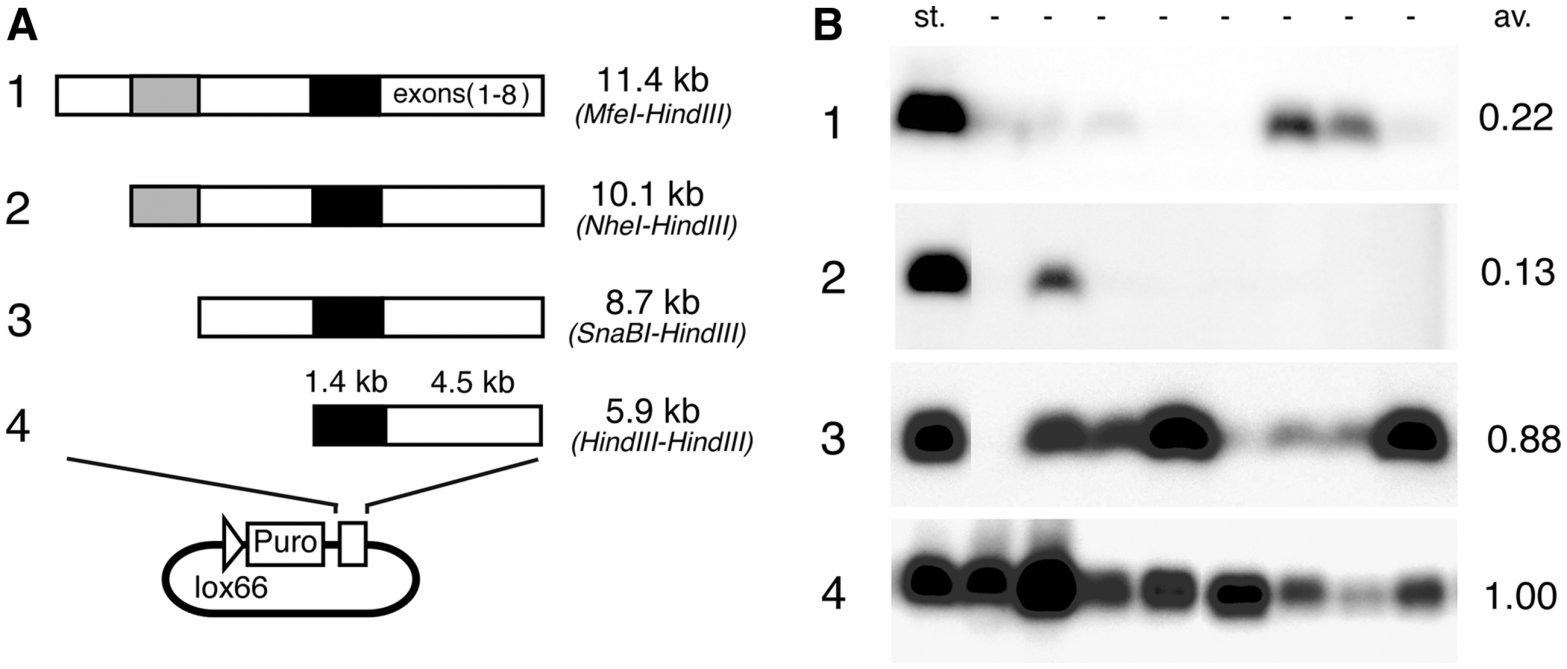
Analysis of HLA-G expression with the HAC vector. (**A**) Four constructs of genomic fragments (1–4) comprised of upstream sequences and HLA-G exons were cloned into the entry vector, then inserted into a HAC vector in HT1080 cells by Cre/lox recombination. Black boxes indicate the reported 1.4-kb promoters; grey boxes indicate the gL1 sequence. (**B**) HLA-G products expressed from four constructs (eight cell lines per construct) were detected by western blot. Lane st. showed the standard product from the 5.9-kb construct (4) for estimating the relative expression level. The average score (av.) was calculated from eight cell lines, with lane st. as the standard.

We investigated four constructs of HLA-G genomic fragments containing the eight exons of HLA-G and associated upstream regions to identify the HLA-G regulatory element (Figure 2A). HLA-G protein was detected via western blot of stable cell lines (eight cell lines per series). Despite the presence of the tissue-specific promoter (10), HLA-G expression driven by the 1.4-kb promoter in a 5.9-kb fragment was not repressed but was fully expressed in non-placental HT1080 cells (Figure 2B).

HLA-G expression from longer genomic fragments was compared with that from the standard 5.9-kb genomic HLA-G sequence. The average ratios were 0.22, 0.13 and 0.88 from the 11.4 kb, 10.1 kb and 8.7 kb regions, respectively (Figure 2B). We reasoned that the remarkable difference in expression levels between the 8.7 kb and 10.1 kb HLA-G fragments were due to a repressive element present in fragments 10.1 kb or longer. Sequence analysis indicated that the region between 8.7 and 10.1 kb did not contain any known elements essential to gene expression, but this region harboured part of a LINE1 element (8,9,15) (gL element; Figure 1A).

### Gene silencing by the gL1 sequence

**Figure 3.**
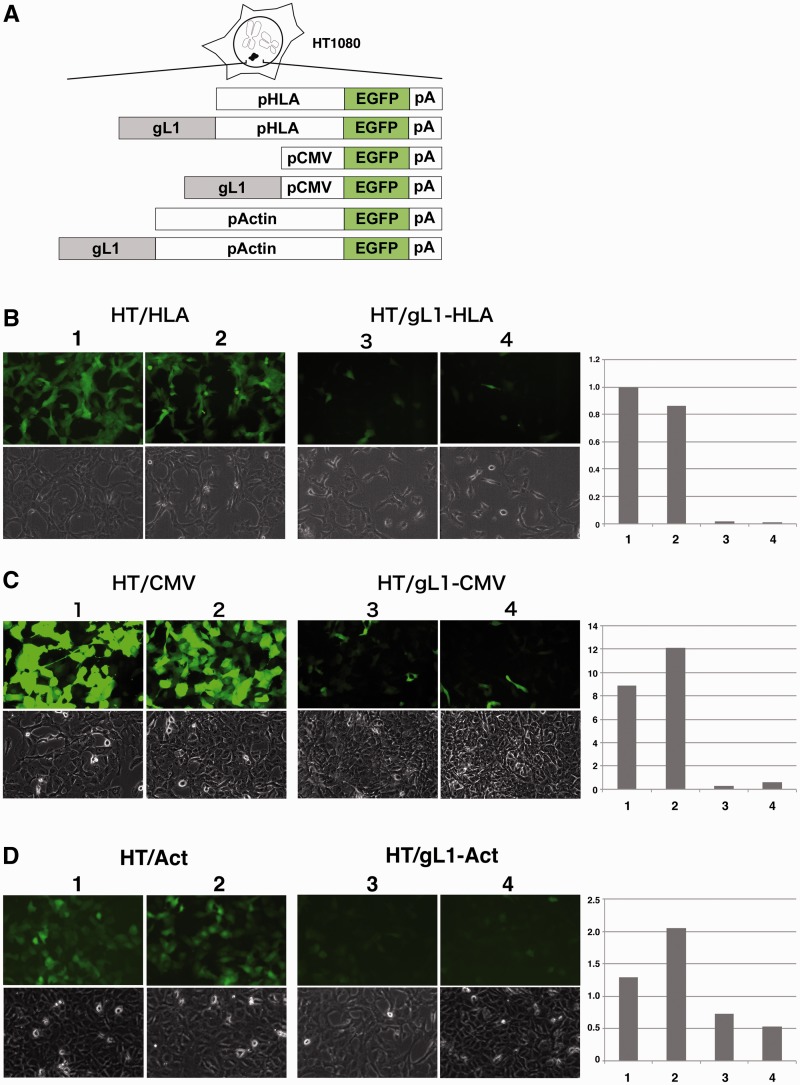
Gene silencing by the gL1 sequence. (**A**) The silencing effect was investigated in HT1080 cells using promoter-driven EGFP reporter gene expression cassettes (HLA-EGFP, CMV-EGFP or Act-EGFP) and cassettes fusing the 1.4 kb gL1 fragment to the HLA, CMV or actin promoters (gL1-HLA-EGFP, gL1-CMV-EGFP or gL1-Act-EGFP, respectively). (**B**, **C**, **D**) EGFP expression was detected by imaging of fluorescence; expression was normalized by RT–PCR. Transcripts from HT/HLA cells (1) served as the standard. (B) The HT/HLA (1 and 2) and HT/gL1-HLA (3 and 4) were HT1080 cell lines harbouring the HLA-EGFP or gL1-HLA-EGFP constructs, respectively. (C) The HT/CMV (1 and 2) and HT/gL1-CMV (3 and 4) were HT1080 cell lines carrying the CMV-EGFP or gL1-CMV-EGFP constructs, respectively. (D) The HT/Act (1 and 2) and HT/gL1-Act (3 and 4) were HT1080 cell lines harbouring gL1-Act-EGFP or Act-EGFP constructs.

To examine whether the 8.7–10.1 kb region of HLA-G possessed a dominant gene silencing element, we created EGFP reporter constructs driven by an HLA-G minimal promoter with or without the 1.4 kb fragment in the LINE1 insertion (SnaBI-NheI: gL1) at the 5′ position (gL1-HLA-EGFP and HLA-EGFP; Figures 1A and 3A). These reporter cassettes were investigated using an HAC vector in HT1080 cells (HT/HLA and HT/gL1-HLA; Figure 3A). The level of EGFP fluorescence from stable cell lines indicated that the standard HLA-EGFP construct was expressed in nearly all cells at uniform levels; however, when fused to the gL1 sequence, EGFP expression was partially or completely repressed in HT/gL1-HLA cells (Figure 3B). Real-time RT–PCR revealed that the level of EGFP transcript in HT/gL1-HLA cells decreased to 14% of that in the HT/HLA cells (Figure 3B).

**Figure 4.**
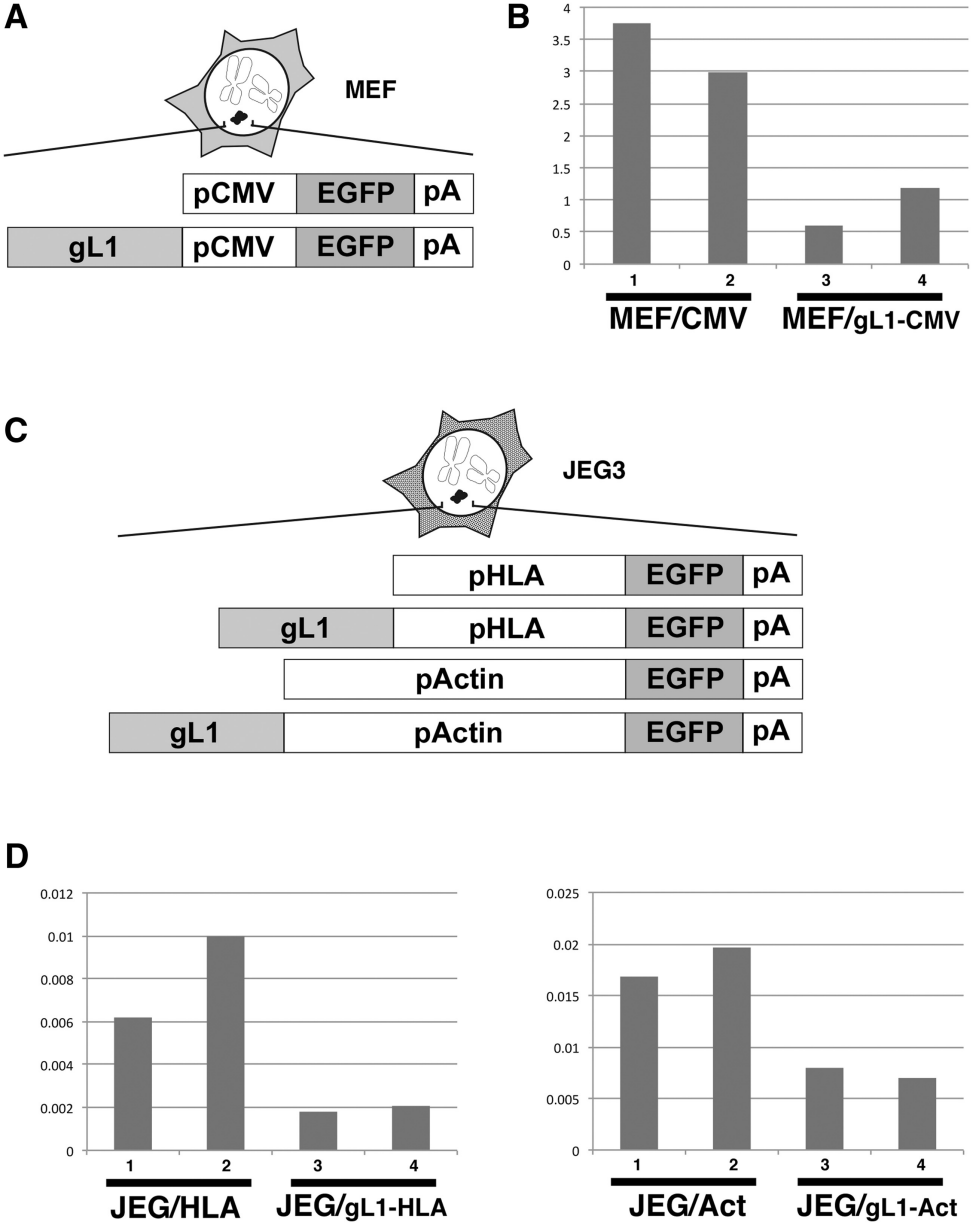
Gene silencing in MEF and JEG3 cells. (**A**) EGFP reporter cassettes (CMV-EGFP) and cassettes fused with the gL1 fragment (gL1-CMV-EGFP) were investigated in MEF cells. (**B**) EGFP expression was detected by RT–PCR. The MEF/CMV (1 and 2) and MEF/gL1-CMV (3 and 4) were MEF cell lines carrying CMV-EGFP or gL1-CMV-EGFP. (**C**) The EGFP reporter cassettes (HLA-EGFP and Act-EGFP) and cassettes fused with the gL1 fragment (gL1-HLA-EGFP and gL1-Act-EGFP) were monitored in JEG3 cells. (**D**) EGFP expression was detected by RT–PCR. The JEG/HLA (1 and 2) and JEG/gL1-HLA (3 and 4) came from JEG3 cell lines carrying HLA-EGFP or gL1-HLA-EGFP, respectively. The JEG/Act (1 and 2) and JEG/gL1-Act (3 and 4) were derived from JEG3 cell lines harbouring the Act-EGFP or gL1-Act-EGFP constructs, respectively.

To examine whether the gL1 sequence was specific or universal to promoters or cell lines, we replaced the HLA-G minimal promoter with the CMV promoter or the human β-actin promoter (Figure 3A). When the gL1 sequence was fused to the CMV or β-actin promoter, the level of EGFP expression in HT1080 cells also decreased to 4% and 37% of that in the cell lines without gL1, respectively (Figure 3C and D). Next, we evaluated MEF cells and JEG3 cells that possessed the HAC vector. Expression from the CMV promoter decreased to 27% of that in the cell lines without gL1 in MEF cells (MEF/gL1-CMV) (Figure 4A and B) and expression from the HLA promoter or the actin promoter decreased to 27% and 41%, respectively, in JEG3 cells (JEG3/gL1-HLA and JEG3/gL1-actin, respectively) (Figure 4C and D).

Taken together, these observations indicated that the gL1 sequence exhibited gene silencing activity under the control of the HLA-G, CMV and actin promoters in HT1080, MEF and JEG3 cells. However, the silencing level varied among promoters and cell lines (Figures 3 and 4). The expression level of all EGFP reporters in JEG3 cells was low by two orders of magnitude compared with that in HT1080 or MEF cells.

### An essential element for gene silencing

**Figure 5.**
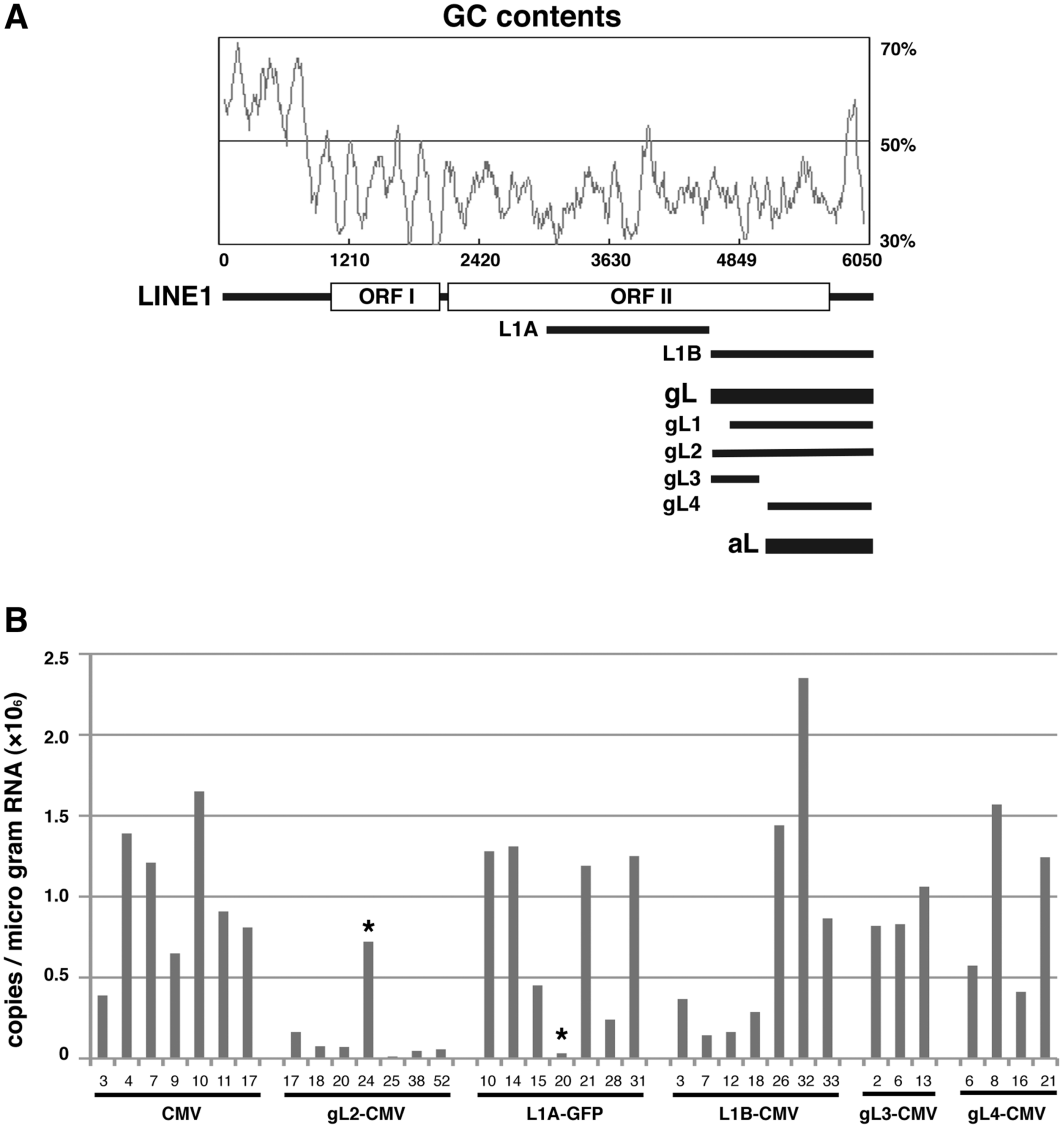
Effects of the LINE1 sequences on gene silencing. (**A**) Configuration and GC contents of segments of the LINE1 sequences. The gL and aL insertions are 1553 bp and 1005 bp segments of the LINE1 sequence found upstream of the HLA-G and HLA-A genes, respectively. L1A and L1B are 1531 bp (position 3010–4540) and 1442 bp (position 4537–5978) segments of the LINE1 family member LIRE1 (6050 bp, M80343). gL1, gL2, gL3 and gL4 are segments of the gL sequence. (**B**) Histograms of normalized EGFP transcript levels detected by RT–PCR (copies/µg RNA). HT1080 cells carried CMV-EGFP (CMV; *n* = 7 cell lines), gL2-CMV-EGFP (gL2-CMV; *n* = 7), L1A-CMV-EGFP (L1A-CMV; *n* = 7), L1B-CMV-EGFP (L1B-CMV; *n* = 7), gL3-CMV-EGFP (gL3-CMV; *n* = 3), or gL4-CMV-EGFP (gL4-CMV; *n* = 4). Cells harbouring the CMV-EGFP construct were used as a control. The asterisk-marked cell lines were exceptional in terms of EGFP expression.

To identify the elements essential for gene silencing, we compared the sequence 8 kb upstream of HLA-G to the sequence upstream of HLA-A; the HLA-A expression was not repressed in most tissues. Another LINE1 sequence was found in the upstream region of HLA-A (aL; Figures 1A and 5A). The alignment with a 6-kb LINE1 sequence (L1RE1) revealed that the gL element was 1553 bp insertion, and the aL element was 1005 bp insertion of non-Ta subfamily of human LINE1 sequence (16,17) (Supplementary Figure S1).

To determine whether the general LINE1 sequence exhibited gene silencing activity, the complete 1.5 kb gL element in HLA-G (gL2; longer than gL1), the corresponding sequence in L1RE1 (L1B) and the 1.5 kb fragment next to L1B in L1RE1 (L1A) were fused to the CMV-EGFP reporter and assayed for gene silencing in HT1080 cells (Figure 5A). EGFP expression from HT1080 cell lines (gL2-CMV, L1A-CMV and L1B-CMV) was compared with that of the control CMV-EGFP cell lines (CMV; Figure 5B). The average levels of EGFP transcripts from the L1A-CMV and L1B-CMV cell lines were similar to that of the control cell lines. The gL2 sequence specifically silenced gene expression driven by the CMV promoter (Figure 5B).

To determine which portion of the gL2 sequence conferred the silencing function, we divided the gL2 sequence into the 5′ (gL3) and 3′ halves (gL4) and assayed CMV-EGFP expression. Neither gL3 nor gL4 repressed CMV-EGFP expression; cell lines harbouring these constructs had EGFP expression levels similar to control CMV-EGFP levels (gL3-CMV and gL4-CMV; Figure 5B). Thus, gene silencing required all parts of the gL element (gL1 or gL2) that were part of the LINE1 sequence, with variations from the L1RE1 sequence.

**Figure 6.**
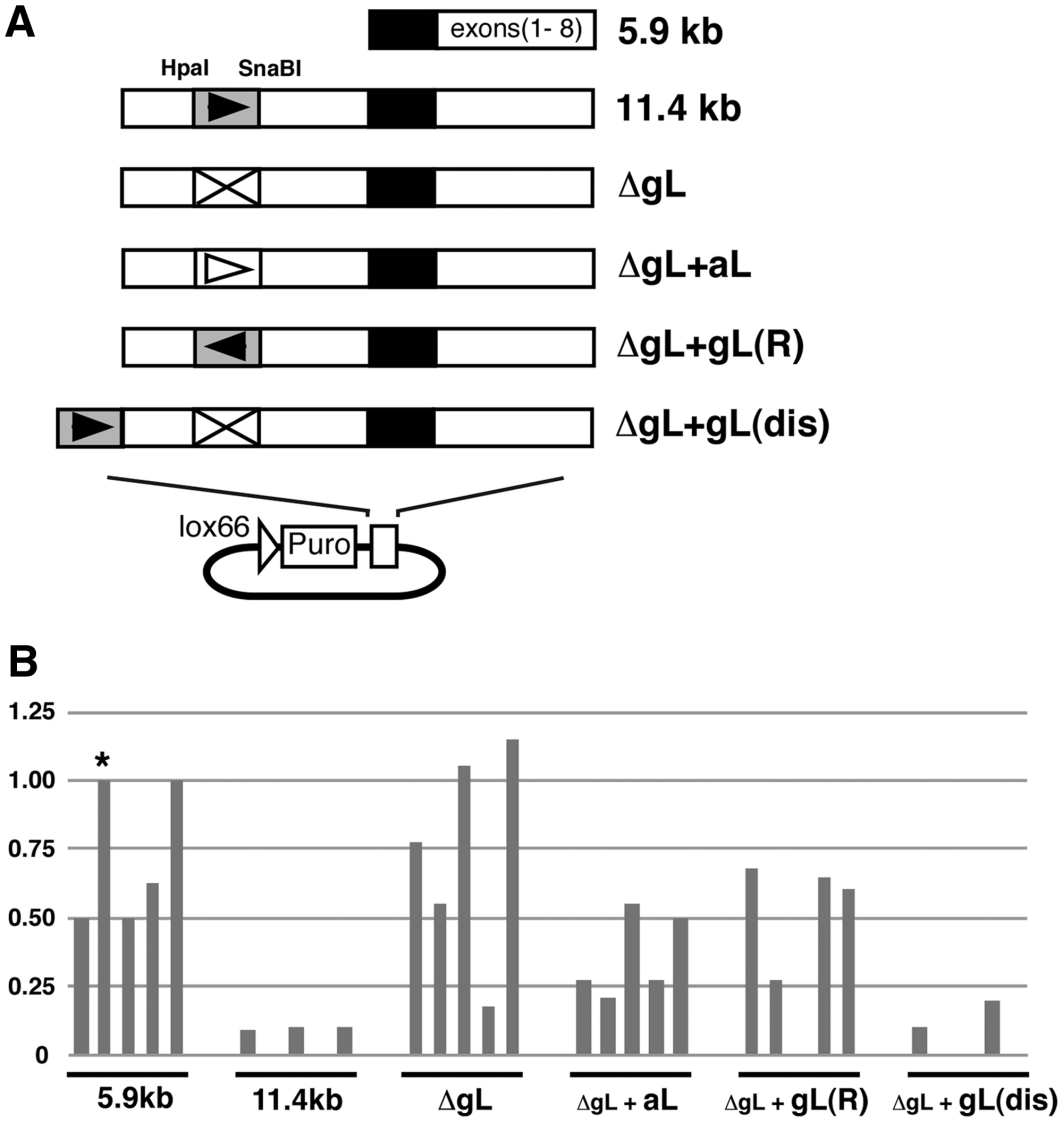
Effects of the gL element in the HLA-G gene. (**A**) Derivatives of HLA-G gene constructs. ΔgL: deletion of gL element (HpaI–SnaBI) from HLA-G (11.4 kb construct). ΔgL+aL: addition of aL to ΔgL. ΔgL+gL(R): addition of gL to ΔgL in an inverted direction. ΔgL+gL(dis): addition of gL to ΔgL at a distal position. (**B**) Histograms of normalized HLA-G transcript levels detected by RT–PCR. The cell lines were HT1080 harbouring HLA-G (5.9 kb), HLA-G (11.4 kb), ΔgL, ΔgL+aL, ΔgL+gL(R), or ΔgL+gL(dis). The cell line carrying HLA-G (5.9 kb; asterisk) was used as a standard.

We inspected the gene silencing function of the gL element in genomic HLA-G region via four series of HLA-G genomic fragments (Figure 6A): a fragment that was deleted for the gL element from the 11.4-kb fragment (ΔgL), ΔgL plus the aL element (ΔgL+aL), ΔgL plus the gL element in the opposite direction [ΔgL+gL(R)] and ΔgL with the addition of the gL sequence at a distal site [ΔgL+gL(dis)]. HLA-G expression from these four series of fragments was measured by real-time RT–PCR and compared with expression from the 11.4-kb (negative control) and 5.9-kb HLA-G fragments (positive control, asterisk: standard cell line; Figure 6B). The average ratios of HLA-G expression for ΔgL, ΔgL+aL and ΔgL+gL(R) (0.74, 0.36 and 0.44, respectively) were highly relative to the positive control (0.73), and the ratio of ΔgL+gL(dis) expression (0.059) was as low as the negative control (0.059). We concluded that the gL element in HLA-G exhibited its silencing function when located at an upstream position (2.8 kb or 5.5 kb from the minimal promoter) and in the forward direction.

### Participation of a hairpin loop in gene silencing

**Figure 7.**
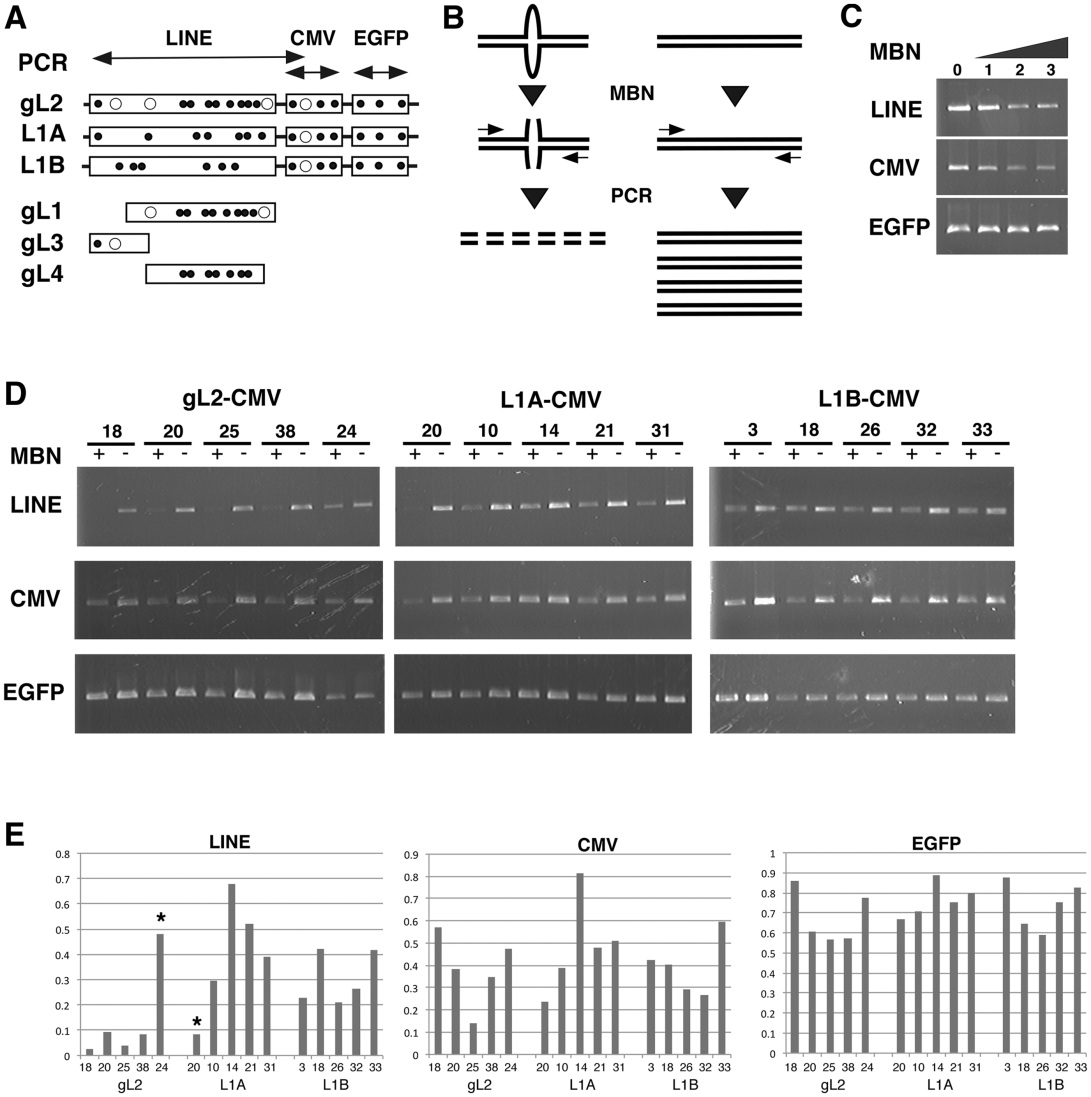
Hairpin structures for gene silencing. (**A**) The positions of potential hairpin loops in the EGFP expression cassette (LINE-CMV-EGFP) were determined based on scores >80% (open circles) or 70–80% (closed circles) in the GENETYX software. Two-way arrows indicate positions for PCR. (**B**) MBN cleaves single-strand DNA that include hairpin loops. The presence of single-strand DNAs was estimated by PCR. (**C**) Conditions leading to MBN cleavage. The genomic DNA from cell line gL2-EGFP20 was cleaved to three levels of MBN (1–3), then amplified by PCR. (**D**) The frequency of hairpin loops was scored by determining the quantity of DNA measured before and after MBN cleavage. Genomic DNA from cell lines harbouring gL2-CMV, L1A-CMV and L1B-CMV constructs (*n* = 5 cell lines each) were amplified before (−) and after (+) MBN cleavage with primers that targeted the LINE elements (gL2, L1A and L1B), the CMV promoter and the EGFP sequence. (**E**) Histograms of the ratios of PCR products of DNA amplified after or before MBN cleavage (MBN +/−). A low ratio indicates a high probability or high frequency of hairpin loops. Asterisks correspond to exceptional cell lines in Figure 5B.

Most of the LINE1 sequences, including the gL element, were characteristic in ∼60% AT-rich, however, a database search did not reveal any typical gene silencers in the gL sequence, nor any specific differences between gL and the general LINE1 sequence. Interestingly, the gL sequence possessed more sites with a higher probability of forming hairpin loops than the general LINE sequence (Figures 5A and 7A). Of these, 10 or 12 of the sites with >70% probability of forming hairpin loops were identified in the gL1 and gL2 fragments that were active for gene silencing, whereas these sites did not frequently occur in L1A, L1B, gL3 or gL4 sequences that were negative for gene silencing (Figure 7A). The mechanism of gene silencing may thus involve hairpin loops in addition to recruitment of silencing factors (18,19).

To examine whether a hairpin loop was present in the gL element, and to characterize the potential participation of such a loop in gene silencing, genomic DNA from five cell lines in three reporter series (gL2-CMV, L1A-CMV and L1B-CMV) was treated with MBN, a single-strand-specific DNA and RNA endonuclease that cleaves hairpin loops (Figure 7B). We then amplified three sites in the expression cassettes corresponding to the 1.5-kb LINE, the 0.6-kb CMV promoter and the 0.6-kb EGFP sequence (Figure 7A). Single-strand DNAs that included hairpin loops resulted in a reduced quantity of amplified product, because cleaved sites between the primer annealing sites were not amplified (Figure 7B). Three conditions for MBN digestion of DNA from the gL2-CMV20 cell line showed a gradual degrease in amplification of LINE and CMV sequences (Figure 7C). The second MBN digestion condition was applied in further analyses of cell lines.

The possibility of hairpin loops was scored by calculating the quantity of DNA measured after and before MBN cleavage (MBN+/MBN−; Figure 7D and E). The average scores for the LINE sequences (gL2: 0.145, L1A: 0.394 and L1B: 0.309) and the CMV promoter region (0.383, 0.484 and 0.397) were lower than those observed for EGFP (0.676, 0.763 and 0.737). These scores corresponded to the frequency of predicted sites for hairpin loop formation as revealed by sequence analysis (Figure 7A and E). Lower scores from the LINE and CMV sequences were distinguishable from high scores from the EGFP sequence. The high score for EGFP indicated that the main targets of MBN cleavage were not transient single-strand DNA molecules produced during replication or transcription, but were single strands formed in DNA secondary structures, including hairpin loops. Importantly, the score for the LINE sequence in the gL2 cell lines was lower than the scores from the L1A and L1B cell lines (Figure 7E). The differences in score likely reflect the probability of formation or the number of hairpin loops. The cell lines that were exceptional in terms of EGFP expression (asterisks in Figures 5B and 7E) also exhibited corresponding value in the MBN analysis; a relatively high score (0.481) was measured in the EGFP active cell line (gL2-CMV-24), and a low score (0.084) was measured in the L1A-CMV-20 cell line that did not express EGFP (Figure 7E). Thus, MBN analysis revealed that a hairpin loop was more likely to form in the gL2 sequence than in the L1A, L1B and CMV promoters, and that the gL2 cell lines that were silenced for EGFP expression contained DNA secondary structure including hairpin loop at gL element.

### Formation of hairpin loops in the genome

**Figure 8.**
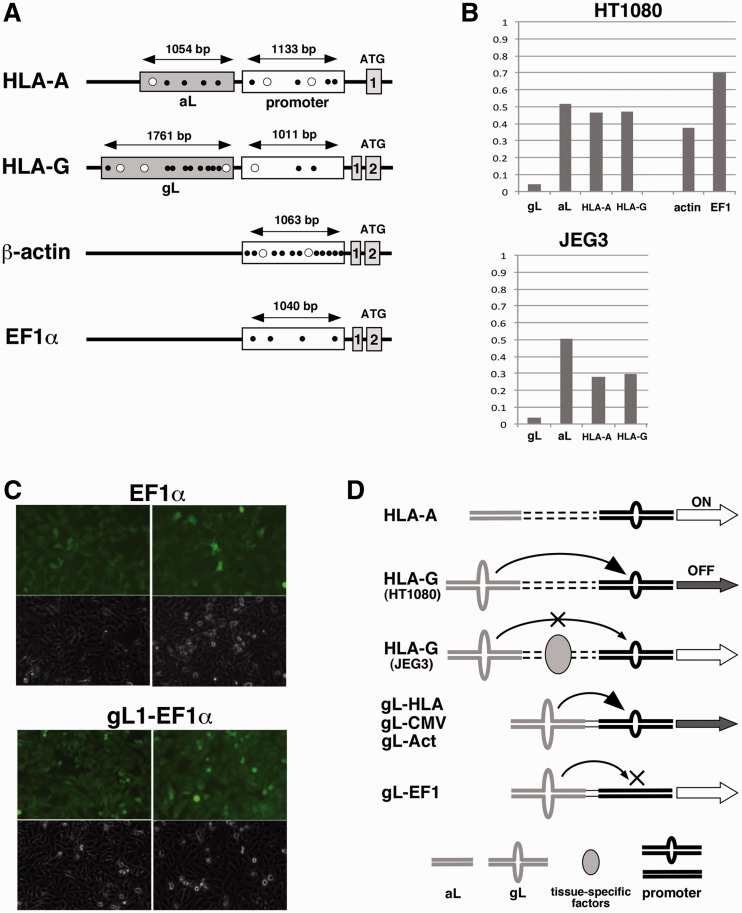
Hairpin structures in the genome. (**A**) Schematic representation of the positions of MBN assay at the promoters and upstream regions of HLA-A, HLA-G, β-actin and EF1α. Grey boxes indicate LINE1 family insertions; open boxes highlight minimal promoter regions. Open circles and closed circles indicate the positions of potential hairpin loops based on scores >80% or >70% (respectively), as determined with the GENETYX software. The positions for PCR amplification are indicated as two-way arrows. PCR primers for LINE1 sequences were set at the boundary between the insertion and the genome. (**B**) Histograms of the probability or frequency of hairpin loops scored by calculating the quantity of DNA measured after and before MBN cleavage (MBN+/−). Genomic DNA from HT1080 and JEG3 cell lines was amplified before and after MBN cleavage with primers that targeted the LINE insertions and the HLA-A, HLA-G, β-actin and EF1α promoter sequences. (**C**) The EF1α promoter-driven EGFP reporter cassette (EF1α) and the cassette containing the gL1 element fused to the EF1α promoter (gL1–EF1α) were investigated in HT1080 cells. EGFP expression was detected by imaging of fluorescence. (**D**) Model of gene silencing mediated by hairpin loops in the genome (HLA-A and HLA-G) or in synthetic constructs (gL-HLA, gL-CMV, gL-Act and gL-EF1). The hairpin loops at the gL elements (grey loops) may react to promoters (black shapes) to trigger gene silencing (arrows). Tissue-specific factors in JEG3 cells may inhibit gene silencing by gL element.

The MBN analysis of the CMV promoter in the EGFP cassettes uncovered the possibility of DNA secondary structures, including hairpin loops, at promoter regions in addition to gL2 sequences (Figure 7E). To understand the mechanism of gene silencing mediated by the gL element, the MBN assay was performed on LINE insertions (gL and aL) and promoter regions in the genomic HLA-A and HLA-G loci (Figure 8A). The MBN score in HT1080 cells was low for the gL element (0.042) and intermediate for the aL, HLA-A, and HLA-G promoter sequences (0.515, 0.467, and 0.473, respectively; Figure 8B). The promoters of the constitutive genes β-actin and EF1α were also investigated in HT1080 cells as controls for the MBN assay in the genomic context. The MBN score was intermediate at the actin promoter (0.378) and high at the EF1α promoter (0.699; Figure 8B). The scores from sequences on the genome corresponded to the predicted frequency of hairpin formation: low scores for gL elements and high scores for EF1α promoters (Figure 8A). Intermediate scores for the HLA-A, HLA-G and β-actin promoters were distinguishable from the high scores of the EF1α promoters. Therefore, the HLA-G promoter in HT1080 cells retains a target for the hairpin loops in the gL element that is absent from the EF1α promoter (Figure 8D).

To confirm the target specificity of the gL element, gene silencing was examined using a reporter cassette (gL1 fused to EF1α-EGFP; gL1-EF1α) in HT1080 cells. EGFP expression from gL1-EF1α was not silenced relative to that from EF1α (Figure 8C), indicating that the EF1α promoter is not a target of the gL element. Thus, the gL element forms a hairpin loop to specify target promoters for silencing.

## DISCUSSION

We investigated the genomic regulation of HLA-G using a HAC vector, identifying a negative regulator of gene expression in a sequence upstream of HLA-G that overlapped with a LINE1 sequence (gL element). LINE1 insertions are abundant in the human genome, therefore, LINE1s with different lengths, sequences or directions were also found within 10 kb upstream of the promoters of HLA-A, HLA-B and HLA-C, but these loci were not silenced. The gene silencing by gL element may be characteristic for HLA-G expression. A mechanism for transcriptional disruption by LINE1 insertion was previously demonstrated that poor LINE1 expression caused inadequate transcriptional elongation of genes, when the LINE1 was inserted inside the transcriptional region (e.g. intron) of the genes (20). However, the insertion length of gL element was 1.5 kb and the position of insertion was upstream of HLA-G promoters.

We demonstrated that a hairpin loop can form in the gL element, and may participate in the silencing of HLA-G (Figures 7 and 8). The correlation of gene silencing with the presence of a hairpin loop was demonstrated in EGFP reporters fused with or without gL element (Figure 7), and in changes of gL2-24 and L1A-20 cell lines, which were from EGFP-active to EGFP-repressed and repressed to active, respectively (Supplementary Figure S2).

We deduced the presence of a hairpin loop at the gL element via MBN analysis, which also revealed potential structural differences between the promoters; CMV, HLA-A, HLA-G and β-actin promoters were associated with intermediate scores, whereas the EF1α promoter received a high score in this analysis (Figure 8). Therefore, gene silencing by the gL element may be characteristic for target promoters. The gL element effectively silenced the native HLA-G promoter, a synthetic CMV promoter and the actin promoter in HT1080 cells, but the gL element did not silence the EF1α promoter in the reporter construct gL1-EF1α (Figure 8C).

Hairpin loops at the gL element may interact with target promoters directly or indirectly to prevent the loading of transcription factors or enhancer factors onto promoters, in addition to the activity of conventional repressors (21). In this model, a hairpin at the gL element may be a highly reactive trigger of gene silencing (Figure 8D). The specificity of silencing may depend not only on the structure of the loop, but also on its sequence, because the gL element lost its silencing activity when placed in a reversed orientation relative to the HLA-G promoter (Figure 6).

Although hairpin loops may form all over the genome, the MBN analysis revealed a much higher probability of hairpin formation on the gL element than on other sequences. Surrounding sequences or chromatin architecture may supply the conditions necessary for hairpin formation on the gL element. For example, hairpin formation may be facilitated by the AT- or A-rich regions in LINE1, which previously exhibited low average nucleosome occupancy both *in vivo* and *in vitro* (22). The hairpin loop may thus provide a simple and flexible trigger for the complex biological process of gene regulation. Despite the presence of hairpin loops on the gL element in JEG3 cells, HLA-G expression was not silenced (Figure 8B). We suggest that HLA-G expression in JEG3 cells may be regulated in a tissue-specific manner that is epistatic to the hairpin-mediated silencing mechanism.

Assuming that HLA-G gene silencing depends on a flexible, DNA structure-based mechanism, we speculate that HLA-G expression may be activated in the foetal placenta or in some tumours via positive regulation. The presence of a LINE1 silencer may explain the limited expression of HLA-G compared with other class I genes.

## SUPPLEMENTARY DATA

Supplementary Data are available at NAR Online: Supplementary Figures 1 and 2.

## FUNDING

MEXT, Japan [Grant-in-Aid for Scientific Research (C) and the Global COE Program for In vivo Human Metabolomic Systems Biology]. Funding for open access charge: Chromo Research Inc.

*Conflict of interest statement*. None declared.

## Supplementary Material

Supplementary Data
