# Supplementary material for: LINE1 family member is negative regulator of HLA-G expression
Source: Nucleic Acids Res. 2012 Sep 21;40(21):10742–52. doi: 10.1093/nar/gks874 (PMC3510505; doi:10.1093/nar/gks874)
Supplement: Supplementary Data [file supp_40_21_10742__index.html]

LINE1 family member is negative regulator of HLA-G expression — LINE1 family member is negative regulator of HLA-G expression — Supplementary Data 

# LINE1 family member is negative regulator of HLA-G expression

## Supplementary Data

files

**Files in this Data Supplement:**

- Supplementary Data - pdf file
