# Supplementary material for: LINE1 family member is negative regulator of HLA-G expression
Source: Nucleic Acids Res. 2012 Sep 21;40(21):10742–52. doi: 10.1093/nar/gks874 (PMC3510505; doi:10.1093/nar/gks874)
Supplement: Supplementary Data [file supp_gks874_nar-01811-v-2012-File010.pdf]

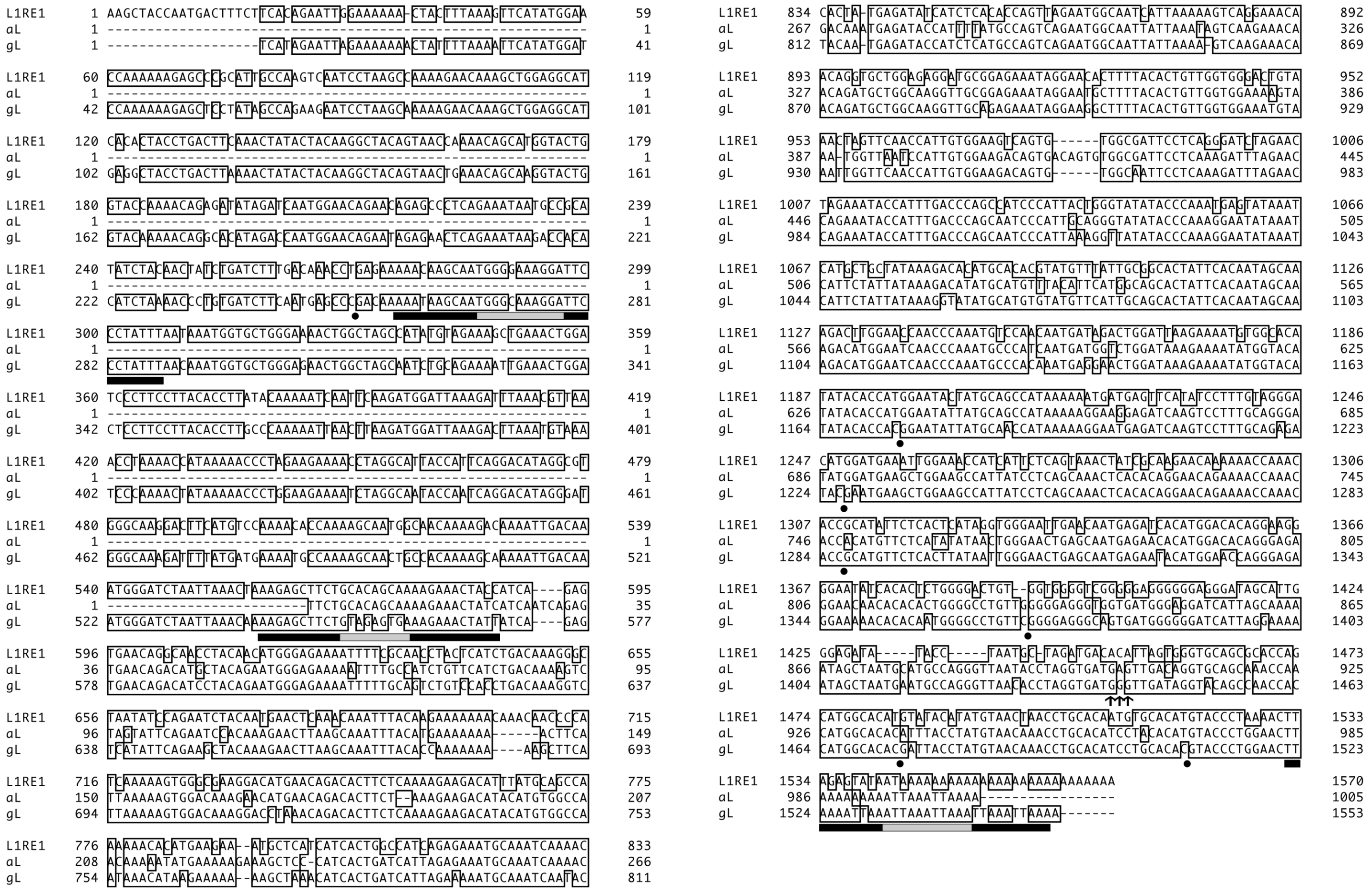

Supplementary Figure 1. Sequence alignment of LINE1 segments.

Alignment of a segment of the L1RE1 (1570 bp), gL (1553 bp), and aL (1005 bp) sequences. Homologous bases are enclosed by frames. Potential hairpin loops (probability above 80%) are shown as bars (black: stem, gray:loop). CpG sequences are shown as small closed circles. Small arrows are position for distinguishing Ta and non-Ta subfamilies.

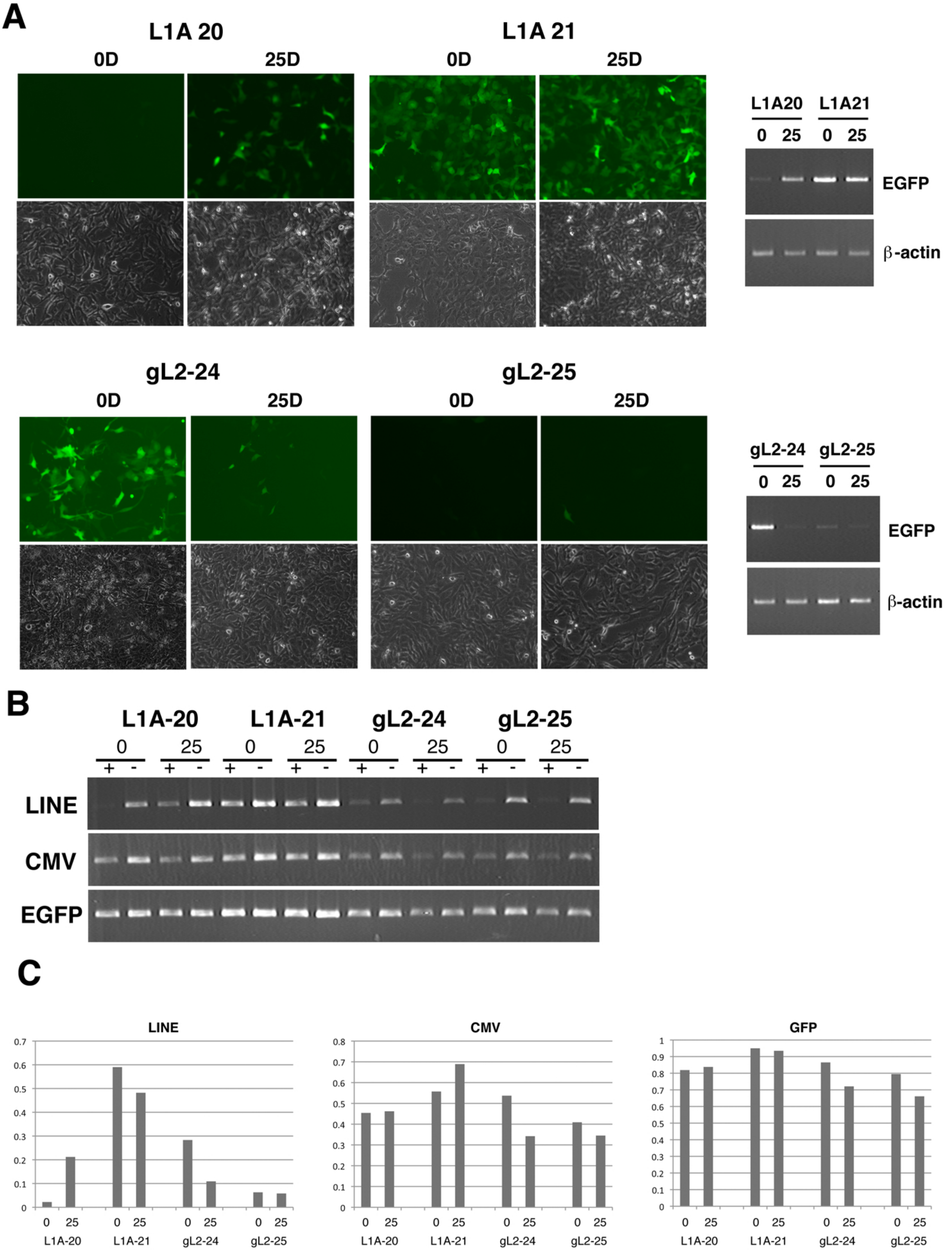

Supplementary Figure 2. Correlation of EGFP expression and MBN score after cell culturing.

(A) EGFP expression levels (imaging of fluorescence, left; RT-PCR, right) were compared before and after 25 days of culturing cell lines L1A-20, L1A-21, gL2-24, and gL2-25. (B) Genomic DNA harvested before or after 25 days of cell culture (lines L1A-20, L1A-21, gL2-24, and gL2-25) was cleaved with MBN (+), then amplified with primers targeting the LINE elements (gL2 and L1A), the CMV promoter, and the EGFP sequence. (C) Histograms of the ratios (MBN +/-) of PCR products.
